# Meta-Analytic Modeling to Define Decision Thresholds for Cerebrospinal Fluid Heparin-Binding Protein in Healthcare-Associated Ventriculitis and Meningitis

**DOI:** 10.3390/diagnostics16071110

**Published:** 2026-04-07

**Authors:** Hsiang-Yi Hung, Po-An Su, Pei-Chun Lai, Yen-Ta Huang

**Affiliations:** 1 Department of Neurosurgery, Hualien Tzu Chi Hospital, Buddhist Tzu Chi Medical Foundation, Hualien City 970, Taiwan; shura0714@yahoo.com.tw; 2 Institute of Medical Sciences, Tzu Chi University, Hualien City 970, Taiwan; 3 Division of Infectious Diseases, Department of Medicine, Chi-Mei Medical Center, Tainan City 710, Taiwan; suboan0421@gmail.com; 4 Education Center, National Cheng Kung University Hospital, College of Medicine, National Cheng Kung University, Tainan City 704, Taiwan; 5 Department of Pediatrics, National Cheng Kung University Hospital, College of Medicine, National Cheng Kung University, Tainan City 704, Taiwan; 6 Department of Surgery, National Cheng Kung University Hospital, College of Medicine, National Cheng Kung University, Tainan City 704, Taiwan

**Keywords:** heparin-binding protein, cerebrospinal fluid, ventriculitis, meningitis, diagnostic accuracy, systematic review, meta-analysis, QUADAS-3, likelihood ratio, neurosurgery

## Abstract

**Background/Objectives:** Healthcare-associated ventriculitis and meningitis (HAVM) is a life-threatening complication of neurosurgical procedures. Conventional cerebrospinal fluid (CSF) indices cannot reliably distinguish bacterial infection from sterile postoperative inflammation, and cultures are frequently delayed or negative. We conducted the first systematic review and meta-analysis to determine the pooled diagnostic accuracy of CSF heparin-binding protein (HBP) for HAVM and to establish clinically actionable decision thresholds. **Methods:** PubMed, Embase, the Cochrane Library, and China National Knowledge Infrastructure were searched from inception to 15 February 2026. Risk of bias was assessed using QUADAS-3. Sensitivity and specificity were pooled with a bivariate random-effects model, and heterogeneity was explored through subgroup analyses and metaregression. Thresholds were derived using likelihood ratio (LR)-based and diagmeta cutoff modeling. **Results:** Twelve studies (*n* = 1761) were included. Pooled sensitivity was 0.861 (95% confidence interval [CI]: 0.777–0.917) and specificity was 0.848 (95% CI: 0.781–0.897), with a positive LR (LR+) of 5.65 and a negative LR (LR−) of 0.164. At a 50% pretest probability, post-test probability was increased to 85% by a positive result and reduced to 14% by a negative result. Intracerebral hemorrhage cohorts showed lower accuracy (sensitivity: 0.675, specificity: 0.755), whereas brain tumor-predominant cohorts demonstrated the highest performance (sensitivity: 0.935, specificity: 0.922; *p* = 0.017). Thresholds of ≥41.3 (rule-in; LR+ ≥10) and ≤30.1 ng/mL (rule-out; LR− ≤0.1) defined clinically actionable decision zones. **Conclusions:** CSF HBP provides quantitatively defined rule-in and rule-out thresholds that meaningfully shift the post-test probability and may support antimicrobial decision-making in suspected HAVM. Prospective multicenter validation is warranted.

## 1. Introduction

Healthcare-associated ventriculitis and meningitis (HAVM) is a severe complication following neurosurgical procedures and external ventricular drain (EVD) insertion, with incidence rates of 4–17 per 1000 catheter-days [1,2]. Its mortality approaches 30%, and up to 62% of HAVM survivors experience persistent neurological impairment [3]. These patients invariably require intensive care unit management. However, the timely and accurate diagnosis of HAVM remains a major clinical challenge.

Postneurosurgical patients commonly develop sterile inflammation with cerebrospinal fluid (CSF) abnormalities clinically indistinguishable from bacterial infection [4]. CSF culture, the current reference standard, demonstrates an overall positivity of approximately 55%, which decreases to 49% with prior antibiotic treatment, and requires 48–72 h to complete [5]. The 2017 Infectious Diseases Society of America (IDSA) guideline states that conventional CSF parameters show limited diagnostic utility [6]. Although the same guideline acknowledges that elevated CSF lactate and procalcitonin may serve as adjunctive markers (weak recommendation, moderate evidence), both are subject to false elevation in the setting of cerebral ischemia, intraventricular hemorrhage, and surgical trauma [6], limiting their discriminatory value in the patients for whom biomarker guidance is most needed.

Heparin-binding protein (HBP) is a neutrophil-derived protein that is rapidly released in response to bacterial pathogens during early infection [7,8]. In contrast to that of traditional inflammatory markers, the elevation of HBP appears to reflect bacterial infection rather than sterile inflammation [7,9] and may not be markedly affected by prior antibiotic administration [10]. Initial studies on community-acquired bacterial meningitis reported high diagnostic accuracy [9], and subsequent neurosurgical studies also demonstrated the promising diagnostic accuracy of CSF HBP for HAVM [10,11].

Despite growing evidence, substantial heterogeneity in reported accuracy and cutoff values across predominantly single-center studies limits the clinical implementation of CSF HBP. We therefore conducted the first systematic review and meta-analysis to determine the pooled diagnostic accuracy of CSF HBP for HAVM, identify sources of heterogeneity, and establish clinically actionable thresholds.

## 2. Materials and Methods

### 2.1. Protocol and Registration

We conducted and reported this systematic review and meta-analysis in accordance with the statement of the Preferred Reporting Items for Systematic Reviews and Meta-Analyses for Diagnostic Test Accuracy Studies (PRISMA-DTA) [12]. The review protocol was prospectively registered in INPLASY (Registration Number: INPLASY202620068; https://doi.org/10.37766/inplasy2026.2.0068), and the PRISMA-DTA checklist is provided in Table S1 from Supplementary Materials.

### 2.2. Eligibility Criteria

We included prospective and retrospective observational studies and randomized controlled trials evaluating the diagnostic accuracy of CSF HBP for HAVM without restrictions on language or patient age. We excluded case reports, reviews, editorials, and conference abstracts.

Studies enrolling patients with suspected HAVM following neurosurgical procedures, traumatic brain injury, or EVD placement were eligible. Studies focusing exclusively on community-acquired meningitis were excluded. The index test was CSF HBP measured by any quantitative assay. The reference standard included any clearly defined diagnostic criteria, whether based on microbiological confirmation; composite clinical and laboratory criteria; established guidelines, such as those from the IDSA; or any combination thereof. Eligible studies were required to report sufficient data to construct 2 × 2 contingency tables.

### 2.3. Information Sources and Search Strategy

Two authors independently searched PubMed, Embase, the Cochrane Library, and China National Knowledge Infrastructure (CNKI) from inception to 15 February 2026. The search strategy combined Medical Subject Headings terms and free-text keywords related to four concepts: heparin-binding protein or azurocidin, ventriculitis/meningitis, healthcare-associated or postneurosurgical context, and CSF. The complete search strategy is provided in Table S2. We manually screened the reference lists of included articles and relevant reviews.

### 2.4. Study Selection and Data Extraction

Two authors independently screened the titles and abstracts, and the full texts of potentially eligible studies were assessed independently. Inter-reviewer agreement was assessed at each stage, with any disagreement resolved by discussion and, when necessary, adjudication by the corresponding authors. Study selection was documented using a Preferred Reporting Items for Systematic Reviews and Meta-Analyses 2020 flow diagram to illustrate identification, screening, eligibility assessment, and inclusion.

Data were independently extracted by two reviewers employing a standardized spreadsheet. Extracted information included study characteristics, population characteristics, assay methods, cutoff values, and reference standard criteria. Diagnostic accuracy data (true positives, false positives, true negatives, and false negatives) were extracted directly or calculated from reported sensitivity, specificity, and sample sizes. Discrepancies were resolved by consensus with the corresponding authors.

### 2.5. Quality Assessment

We assessed methodological quality with the Quality Assessment of Diagnostic Accuracy Studies-3 (QUADAS-3) tool [13]. QUADAS-3 employs a six-phase assessment: Phases 1–2 define the review question and ideal study characteristics, whereas Phases 3–6 evaluate each study’s risk of bias across four domains (Participants, Index Test, Target Condition, and Analysis) and applicability concerns across three domains (Participants, Index Test, and Target Condition). Overall judgments were determined as high if any domain was high, low if all domains were low, and insufficient if any domain had insufficient information and none were high. Prior to independent assessment, the two reviewers calibrated their application of QUADAS-3 signaling questions through discussion to ensure consistent interpretation. The two reviewers then independently completed all phases, with disagreements resolved by the corresponding authors.

### 2.6. Statistical Analysis

We organized diagnostic accuracy data from each study into 2 × 2 contingency tables. We calculated pooled estimates of sensitivity and specificity with 95% confidence intervals (CIs) using a bivariate random-effects model via restricted maximum likelihood estimation [14]. We derived the pooled positive likelihood ratio (LR+), negative likelihood ratio (LR−), and diagnostic odds ratio (DOR) from the pooled estimates.

We conducted subgroup analyses by fitting separate bivariate models for subgroups defined by underlying pathology, study design, assay method, and reference standard stringency. Subgroups with fewer than three studies were not subjected to bivariate modeling. We performed exploratory univariable metaregression by adding each covariate individually to the bivariate model, with significance assessed with the likelihood ratio (LR) test.

Between-study variability was modeled within a bivariate random-effects framework and examined using the summary receiver operating characteristic (SROC) curve with 95% confidence and prediction regions. Cochran’s Q and I^2^ statistics were calculated descriptively for sensitivity and specificity, but were not considered as primary measures of heterogeneity given the hierarchical structure of the diagnostic accuracy meta-analysis. We evaluated threshold effects through the between-study correlation between logit-transformed sensitivity and the logit-transformed false positive rate derived from the bivariate model. We assessed clinical utility using Fagan nomograms at pretest probabilities of 25%, 50%, and 75% to illustrate shifts in post-test probability [15], and we assessed publication bias by employing Deeks’ funnel plot asymmetry test [16].

We performed prespecified optimal cutoff analysis using the method of Steinhauser et al. [17], which models sensitivity and specificity as functions of the cutoff value through weighted least squares regression. Three transformations (linear, logarithmic, and square root) were compared using the combined Akaike information criterion, and the optimal cutoff was defined as the value maximizing the Youden index obtained via bootstrapping. Additional criteria explored included cutoffs maximizing LR+ (rule-in) and minimizing LR− (rule-out). This approach enabled derivation of continuous LR+ and LR− curves across the full range of observed cutoff values, from which clinically actionable thresholds were identified at predefined operating points of LR+ ≥ 10 and LR− ≤ 0.1, thresholds widely considered to produce large and often decisive shifts in post-test probability.

All analyses were conducted in R (Version 4.5.0), leveraging the mada package for bivariate diagnostic meta-analysis and the diagmeta package for cutoff modeling. Statistical significance was set at *p* < 0.05 for all tests, except for Deeks’ test, where *p* < 0.10 was used.

## 3. Results

### 3.1. Study Selection

**Figure 1:**
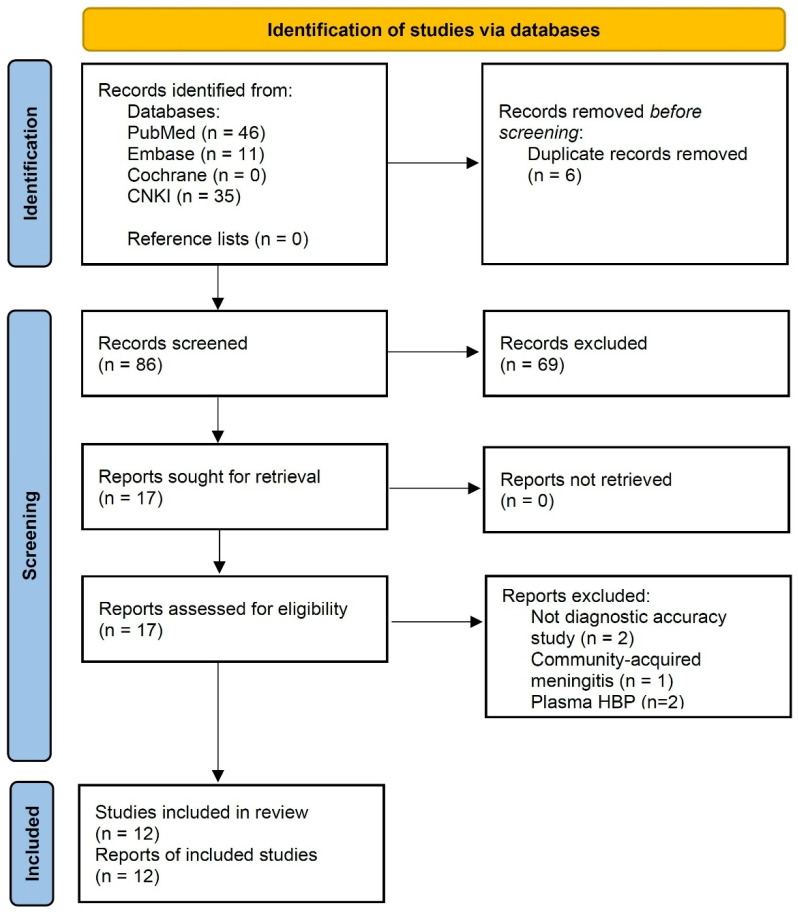
Flow diagram of study identification, screening, eligibility assessment, and inclusion. CNKI, China National Knowledge Infrastructure; HBP, heparin-binding protein.

Our initial search identified 92 records across four databases (PubMed, 46; Embase, 11; Cochrane Library, 0; and CNKI, 35), without additional records obtained from reference list screening. After we removed 6 duplicates, 86 records underwent title and abstract screening. Of these records, we assessed 17 full-text articles for eligibility and excluded 5 for the following reasons: did not report a diagnostic accuracy study (*n* = 2), reported on community-acquired meningitis (*n* = 1), and reported plasma HBP only (*n* = 2). Ultimately, we included 12 studies comprising 1761 patients (Figure 1).

### 3.2. Study Characteristics

The 12 included studies are summarized in Table S3. The studies were published between 2021 and 2025, with sample sizes ranging from 58 to 390 (total of 1761, with 841 infected and 920 noninfected patients). Eleven were conducted in China [10,11,18,19,20,21,22,23,24,25,26], and one in Sweden [27]. All studies were single-center in design. Four employed a prospective or partially prospective design [10,18,26,27], and the remaining eight were retrospective.

Patient populations varied considerably. Three studies exclusively enrolled patients with ICH [19,21,25], two exclusively enrolled patients undergoing brain tumor surgery [18,24], and one predominantly enrolled patients with brain tumors (~76%) [10]. Two studies included mixed neurosurgical populations [26,27], whereas the remaining four did not report the distribution of primary diagnoses [11,20,22,23].

CSF HBP was measured through an enzyme-linked immunosorbent assay (ELISA, *n* = 5), latex immunoturbidimetry (*n* = 3), fluorescence immunoassay (*n* = 2), or fluorescence immunochromatography (*n* = 1). One study did not specify the assay method. Reference standards for HAVM diagnosis included composite clinical and CSF criteria based on Chinese national diagnostic standards (*n* = 6), the 2017 IDSA guidelines (*n* = 2), the surveillance definition provided by the Centers for Disease Control and Prevention/National Healthcare Safety Network (*n* = 1), microbiological confirmation with clinical criteria (*n* = 2), and clinical criteria without further specification (*n* = 1). Comparator groups consisted of postneurosurgical patients without infection (*n* = 9), patients with aseptic meningitis (*n* = 2), or nonbacterial controls (*n* = 1). Reported cutoff values ranged from 14.96 ng/mL to 92.5 ng/mL; two studies did not report specific cutoffs.

### 3.3. Quality Assessment

The QUADAS-3 results are presented in Table S4. Of the 12 included studies, 6 (50%) were judged as having high overall risk of bias, 5 (42%) as low risk, and 1 (8%) as having insufficient information because of the inadequate reporting of index test methodology. High risk of bias was primarily attributed to retrospective two-gate case–control designs in the Participants domain; such designs may lead to spectrum bias and diagnostic accuracy overestimation. The Index Test domain showed low risk across all studies owing to the use of standardized assays. The Target Condition domain was rated as low risk in 10 studies (83.3%), with two studies rated as having insufficient information. The Analysis domain exhibited low risk in all studies. Applicability concerns were generally low. Two studies restricted to ICH-only populations [19,25] raised concerns regarding external validity, because such narrowly defined cohorts may not represent the broad population of patients with suspected HAVM.

### 3.4. Diagnostic Accuracy of CSF HBP

**Figure 2:**
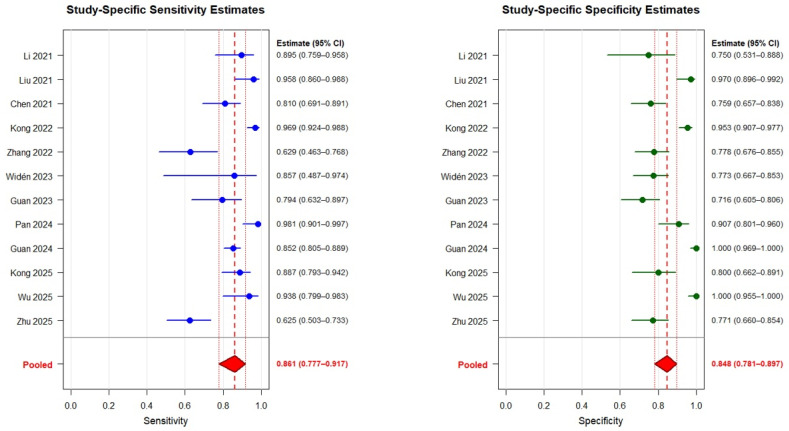
Paired forest plots of study-specific sensitivity (**left**) and specificity (**right**) estimates for cerebrospinal fluid heparin-binding protein in diagnosing healthcare-associated ventriculitis and meningitis. Individual study point estimates and 95% confidence intervals are displayed alongside numerical values. Pooled estimates (red diamonds) were calculated by using a bivariate random-effects model. Red dashed lines indicate pooled estimates; red dotted lines indicate corresponding 95% confidence intervals [10,18,19,20,22,23,24,25,26,27].

**Figure 3:**
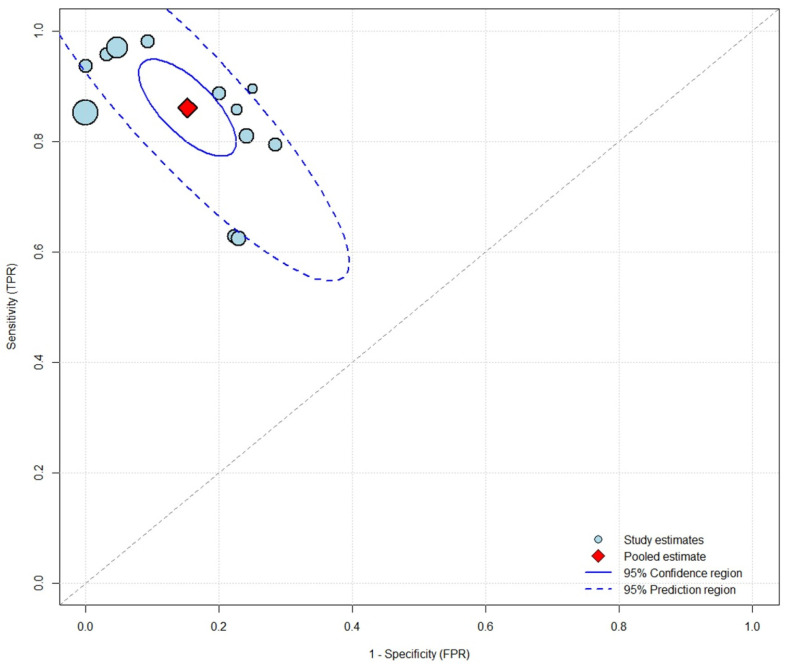
Summary receiver operating characteristic plot for cerebrospinal fluid heparin-binding protein in diagnosing healthcare-associated ventriculitis and meningitis. Each circle represents an individual study, with circle size proportional to sample size. The red diamond indicates the pooled estimate. The solid ellipse represents the 95% confidence region; the dashed ellipse represents the 95% prediction region. The diagonal gray line represents the line of no discrimination. FPR, false positive rate; TPR, true positive rate.

CSF HBP for HAVM diagnosis had a pooled sensitivity of 0.861 (95% CI: 0.777–0.917) and pooled specificity of 0.848 (95% CI: 0.781–0.897) (Figure 2). Individual study sensitivity ranged from 0.625 to 0.981, and specificity ranged from 0.716 to 1.000. The pooled LR+ was 5.65 (95% CI: 3.62–8.75), LR− was 0.164 (95% CI: 0.094–0.280), and DOR was 34.5 (95% CI: 13.3–88.8). Substantial heterogeneity was present for sensitivity (I^2^ = 80.6%) and specificity (I^2^ = 98.5%). A strong negative correlation between logit-transformed sensitivity and the false positive rate (ρ = −0.920) indicated a substantial threshold effect, suggesting that differences in applied cutoff values were an important contributor to heterogeneity. The SROC curve is shown in Figure 3. Most study estimates clustered in the upper-left quadrant of the ROC space, although the wide 95% prediction region reflected the substantial between-study heterogeneity.

### 3.5. Clinical Utility and Publication Bias

**Figure 4:**
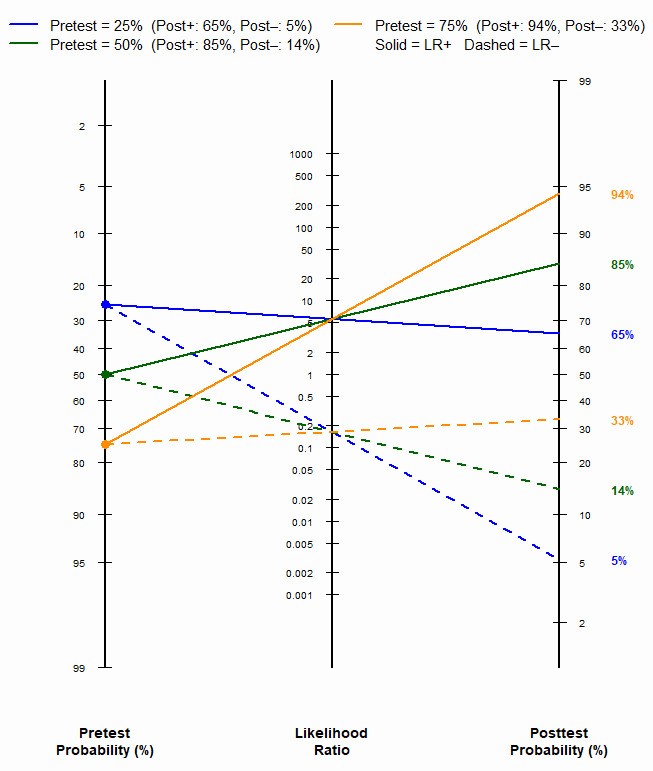
Fagan nomogram illustrating the clinical utility of cerebrospinal fluid heparin-binding protein for diagnosing healthcare-associated ventriculitis and meningitis at three pretest probabilities (25%, 50%, and 75%). Solid lines represent the positive likelihood ratio (LR+); dashed lines represent the negative likelihood ratio (LR−). Posttest probabilities are displayed on the right axis. LR, likelihood ratio.

**Figure 5:**
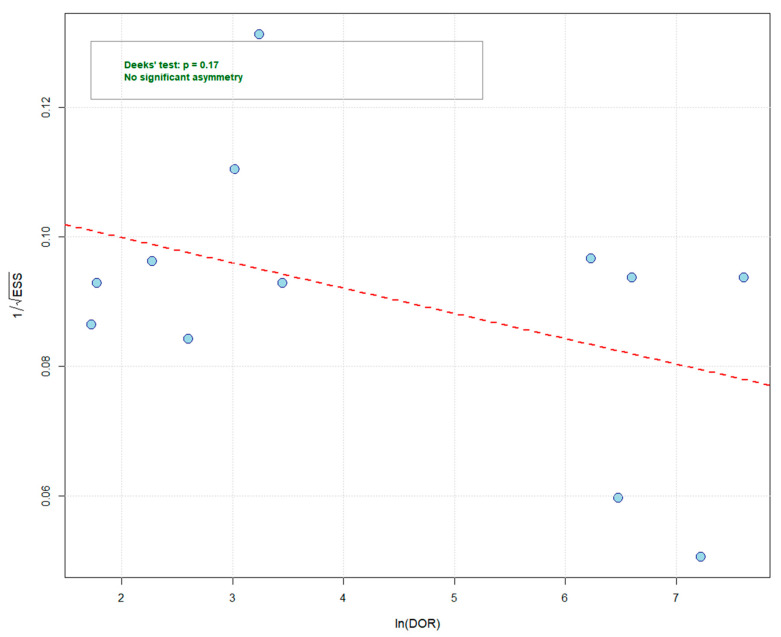
Deeks’ funnel plot for the assessment of publication bias. Each circle represents an individual study, plotted by the natural logarithm of the diagnostic odds ratio against the inverse square root of the effective sample size. The red dashed line represents the regression line. The nonsignificant slope (*p* = 0.17) suggests no substantial publication bias. DOR, diagnostic odds ratio; ESS, effective sample size.

The Fagan nomogram demonstrated that at a pretest probability of 50%, the post-test probability was increased to 85% by a positive CSF HBP result but was decreased to 14% by a negative result (Figure 4). The corresponding post-test probabilities at pretest probabilities of 25% and 75% were 65%/5% and 94%/33%, respectively. Deeks’ funnel plot asymmetry test showed no significant publication bias (*p* = 0.17) (Figure 5).

### 3.6. Subgroup and Metaregression Analyses

Our subgroup analyses revealed that underlying pathology substantially influenced diagnostic performance (Table 1). ICH-predominant studies demonstrated markedly lower pooled sensitivity (0.675; 95% CI: 0.556–0.775) and specificity (0.755; 95% CI: 0.688–0.812) compared with non-ICH studies (sensitivity: 0.900, specificity: 0.872), whereas brain tumor-predominant populations yielded the highest accuracy among populations (sensitivity: 0.935, specificity: 0.922, DOR 170.8). Metaregression confirmed the ICH population as a statistically significant source of heterogeneity (*p* = 0.017).

Diagnostic accuracy was numerically higher in prospective studies than in retrospective studies (sensitivity: 0.896 vs. 0.849; specificity: 0.912 vs. 0.833), in studies using strict standards than in those employing composite reference standards (sensitivity: 0.905 vs. 0.820; specificity: 0.868 vs. 0.830), and in non-ELISA-based studies than in ELISA-based studies (sensitivity: 0.886 vs. 0.788; specificity: 0.865 vs. 0.760). However, none of these covariates reached statistical significance in the metaregression (*p* = 0.565 and *p* = 0.332 for study design and reference standard, respectively), likely reflecting limited power given the small number of studies.

### 3.7. Optimal Cutoff Analysis

Among the 10 studies reporting explicit cutoff values, the cutoff maximizing the Youden index was 28.4 ng/mL (predicted sensitivity: 0.905, specificity: 0.929, LR+: 12.8, LR−: 0.103) (Figures S1 and S2). However, the 95% bootstrap CI was wide (7.5–111.0 ng/mL), reflecting relatively stable Youden index values across approximately 20–50 ng/mL (Youden index > 0.83) (Figure S3). This finding suggests that no single cutoff was statistically superior within this interval and supports the concept of an equivalence range, rather than a uniquely optimal threshold.

**Figure 6:**
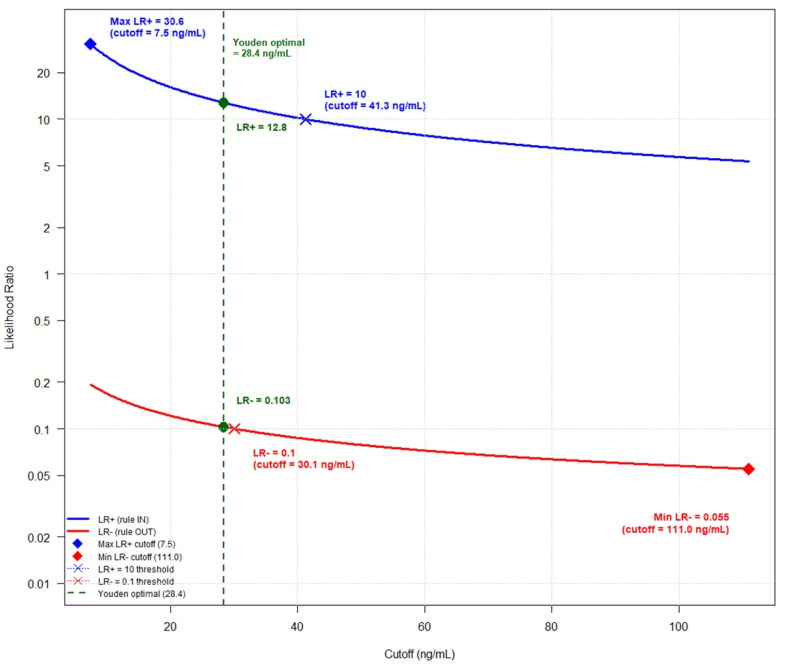
Likelihood ratio curves as a function of cerebrospinal fluid heparin-binding protein cutoff values. The upper blue curve represents the positive likelihood ratio (LR+, rule-in), and the lower red curve represents the negative likelihood ratio (LR−, rule-out). The dashed green line indicates the cutoff maximizing the Youden index (28.4 ng/mL). Clinically relevant operating thresholds are marked: LR+ = 10 at 41.3 ng/mL and LR− = 0.1 at 30.1 ng/mL (crosses). Horizontal dotted lines denote the LR+ = 10 and LR− = 0.1 reference levels. LR, likelihood ratio.

The LR criteria of LR+ ≥ 10 and LR− ≤ 0.1 are widely considered to produce large and often clinically decisive shifts in post-test probability [28]. In the present analysis, these operating characteristics were achieved at approximately 41.3 (rule-in) and 30.1 ng/mL (rule-out) (Figure 6). Although the modeled curves yielded extreme LRs at boundary cutoffs (e.g., maximum LR+ at 7.5 ng/mL and minimum LR− at 111.0 ng/mL), such estimates likely reflect statistical extrapolation beyond the range of empirically observed thresholds rather than clinically validated operating points. Accordingly, interpretation should focus on threshold regions supported by underlying study data.

From a clinical perspective, CSF HBP levels above 41.3 ng/mL would substantially increase the probability of HAVM and may justify prompt antimicrobial initiation, whereas levels below 30.1 ng/mL would markedly reduce post-test probability and could support the consideration of withholding or de-escalating empiric therapy in appropriately selected patients. This includes patients without clinical deterioration, without microbiological evidence of infection, and without high-risk features such as a predominant ICH burden or immunocompromised status.

## 4. Discussion

To our knowledge, this work is the first meta-analysis evaluating CSF HBP for HAVM diagnosis. Our analysis of 12 studies comprising 1761 patients showed that CSF HBP provides clinically meaningful diagnostic performance and produces substantial shifts in post-test probability across clinically relevant pretest probabilities in suspected HAVM, with a pooled sensitivity of 0.861 and a specificity of 0.848. At a pretest probability of 50%, the post-test probability was raised to 85% by a positive result and decreased to 14% by a negative result, indicating that a single CSF HBP measurement can meaningfully shift clinical decision-making in either direction.

Distinguishing bacterial infection from sterile inflammation remains a central diagnostic challenge in postneurosurgical patients, as brain injury and intraventricular hemorrhage can elevate traditional CSF markers [6,29]. Previous meta-analyses reported pooled sensitivities and specificities of 0.82/0.81 for CSF procalcitonin, although with notably wide CIs reflecting substantial heterogeneity [30]. Although CSF lactate demonstrated high pooled estimates (sensitivity: 0.92, specificity: 0.88) [31], this specificity may be misleadingly optimistic because cerebral ischemia and intraventricular hemorrhage independently elevate lactate levels, producing false positives precisely in the population where biomarker guidance is most needed [6]. CSF HBP offers a comparable or potentially superior diagnostic profile to traditional CSF markers, with the additional advantage of reported resistance to antibiotic interference [10].

Substantial between-study heterogeneity was largely attributable to a threshold effect driven by varying cutoff values across studies. Rather than indicating inconsistent intrinsic test performance, this pattern primarily reflects variation in applied decision thresholds and underscores the need for LR-based cutoff modeling. Beyond threshold effects, however, biomarker performance also appeared to be influenced by underlying pathology. Subgroup analysis and metaregression identified underlying pathology as the only statistically significant moderator, with ICH-predominant studies showing markedly lower diagnostic performance than non-ICH studies. This finding is biologically plausible because ICH causes extensive blood–brain barrier disruption and neutrophil product release into the CSF, elevating baseline HBP levels and blurring the distinction between infected and noninfected patients [6,29]. By contrast, brain tumor-predominant studies yielded the highest accuracy among studies, likely reflecting a clean inflammatory baseline. These findings suggest that CSF HBP interpretation should account for underlying pathology and that increased cutoffs or adjunctive biomarkers may be warranted in post-ICH patients.

**Figure 7:**
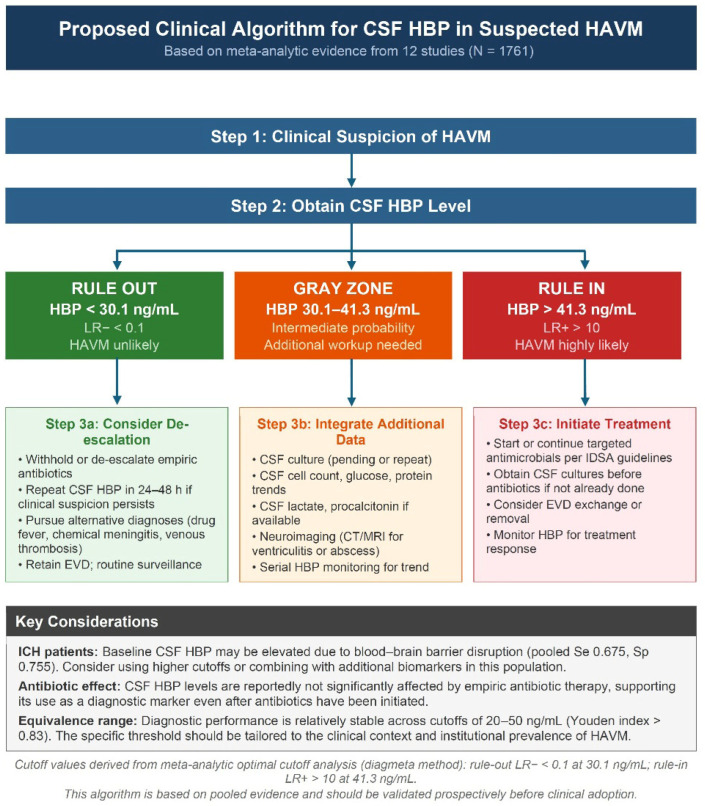
Proposed decision-support framework for integrating cerebrospinal fluid heparin-binding protein into the diagnostic evaluation of suspected healthcare-associated ventriculitis and meningitis. This three-tier model translates meta-analytically derived LR thresholds into a pragmatic clinical workflow. Cerebrospinal fluid heparin-binding protein values less than 30.1 ng/mL (LR− ≤0.1) define a rule-out zone associated with a substantial reduction in post-test probability, potentially supporting antimicrobial de-escalation in appropriately selected patients. Values >41.3 ng/mL (LR+ ≥10) define a rule-in zone associated with large probability shifts favoring early antimicrobial initiation. Intermediate values (30.1–41.3 ng/mL) constitute a gray zone requiring the integration of clinical findings, microbiological data, and underlying pathology, particularly in ICH populations where baseline inflammatory activation may attenuate specificity. This framework is intended to complement, rather than replace, existing diagnostic criteria (e.g., IDSA guidelines) and emphasizes the incorporation of pretest probability assessment in neurocritical care settings. This framework is intended for hypothesis-generating clinical decision support and requires prospective validation. Abbreviations: BBB, blood–brain barrier; CT, computed tomography; EVD, external ventricular drain; ICH, intracerebral hemorrhage; IDSA, Infectious Diseases Society of America; LR+, positive likelihood ratio; LR−, negative likelihood ratio; MRI, magnetic resonance imaging.

Although the optimal cutoff maximizing the Youden index was 28.4 ng/mL, Youden index values remained largely stable across approximately 20–50 ng/mL, indicating that no single cutoff was statistically superior within this range. From an evidence-based medicine perspective, LR+ ≥ 10 and LR− ≤ 0.1 are generally considered to produce large and often clinically decisive shifts in post-test probability [28]. In our present analysis, these thresholds were reached at approximately 41.3 (rule-in) and 30.1 ng/mL (rule-out). Levels above 41.3 ng/mL strongly support HAVM and may justify prompt antimicrobial initiation in appropriate clinical contexts, whereas levels below 30.1 ng/mL substantially reduce the likelihood of HAVM and may support consideration of withholding or de-escalating antimicrobial therapy. In the context of antimicrobial resistance, a quantitatively defined rule-out zone may support antimicrobial stewardship by reducing diagnostic uncertainty and potentially limiting unnecessary exposure to broad-spectrum agents in postneurosurgical patients, in whom empiric therapy is often prolonged. Patients with intermediate values require the integration of additional clinical and laboratory data. The derived thresholds should therefore be interpreted as clinically informative reference points, rather than definitive diagnostic cutoffs. Based on these findings, we propose an evidence-based algorithm integrating CSF HBP into the diagnostic workup of suspected HAVM (Figure 7), incorporating pretest probability assessment, a three-tier decision framework, and corresponding management recommendations, with a caveat for cautious interpretation in patients with ICH.

Notably, the diagnostic thresholds derived in our present analysis (rule-out ≤ 30.1 ng/mL; rule-in ≥ 41.3 ng/mL) are substantially higher than those reported for community-acquired bacterial meningitis. Olie et al. [32] recently demonstrated, in a prospective Dutch multicenter cohort, that a CSF HBP cutoff of 5.2 ng/mL effectively distinguished bacterial meningitis from other central nervous system disorders. This discrepancy in optimal thresholds is expected and can be attributed to fundamental differences in the target population. In community-acquired meningitis, the noninfected comparator group comprised patients with largely intact blood–brain barriers and minimal baseline neuroinflammation, resulting in low CSF HBP levels in noninfected individuals and allowing even modest elevations to achieve diagnostic discrimination. By contrast, postneurosurgical patients uniformly exhibit some degree of blood–brain barrier disruption and surgical trauma-induced neuroinflammation, with ICH further amplifying nonspecific inflammatory responses in the CSF, all of which elevate baseline HBP levels irrespective of infection [6,29]. This higher inflammatory baseline in the HAVM population than in other populations necessitates correspondingly higher thresholds to distinguish true bacterial infection from sterile postoperative inflammation. These findings reinforce the principle that CSF HBP cutoffs are population-specific and should not be extrapolated across distinct clinical settings.

To our knowledge, our study represents one of the first systematic reviews to employ the recently published QUADAS-3 tool for quality assessment. QUADAS-3 offers several methodological advantages over QUADAS-2. The structured six-phase framework requires the explicit definition of an “ideal” diagnostic accuracy study before the assessment of individual studies, promoting transparency and consistency in quality judgments. The expanded signaling questions in the Target Condition domain allow for nuanced evaluation of reference standard adequacy, which is particularly relevant for HAVM diagnosis given the substantial variation in diagnostic criteria across studies. Additionally, the clear decision rules for overall judgments enhance reproducibility. Our assessment identified retrospective two-gate case–control design as the predominant source of a high risk of bias; this finding is consistent with previous observations that such designs tend to overestimate diagnostic accuracy by excluding diagnostically ambiguous patients [33].

The reference standard definitions varied considerably across studies, with at least five different diagnostic criteria used, including the IDSA guideline [6], composite clinical criteria, and microbiological confirmation. Such heterogeneity may introduce the differential misclassification of the target condition and could partially influence pooled diagnostic accuracy estimates. This variability directly affects accuracy estimates and represents a major challenge for cross-study comparison. Heterogeneity in HBP assay platforms represents an additional methodological concern. Five distinct measurement technologies were employed across included studies, including ELISA, latex immunoturbidimetry, fluorescence immunoassay, and fluorescence immunochromatography. These platforms differ in analytical sensitivity, calibration standards, and absolute concentration outputs, which may introduce systematic between-study variability in reported HBP values independent of true biological differences. Although subgroup analysis suggested numerically higher diagnostic accuracy in non-ELISA-based studies compared with ELISA-based studies, this difference did not reach statistical significance, likely reflecting insufficient power rather than true equivalence. Consequently, the derived thresholds of 41.3 and 30.1 ng/mL should be regarded as platform-agnostic reference points pending prospective validation within individual assay systems. Standardization of HBP measurement platforms will be a prerequisite for the clinical implementation of any universal cutoff.

Several additional limitations should be acknowledged. Eleven of the 12 included studies were conducted in single academic centers in China, raising concerns regarding geographic concentration and potential spectrum bias, which may limit the generalizability of the pooled diagnostic accuracy estimates. External validation in diverse healthcare systems is therefore essential before broad clinical adoption. The small number of studies restricts subgroup and metaregression power, and subgroup-specific estimates should be considered hypothesis-generating. Furthermore, the predominance of retrospective single-center designs among included studies introduces additional concerns: retrospective two-gate case–control designs are known to overestimate diagnostic accuracy by excluding diagnostically ambiguous patients [33], and single-center studies may reflect institution-specific patient selection, assay calibration, and clinical thresholds that do not generalize broadly. With only 12 studies available, the bivariate model operates near the lower boundary of stability, and pooled estimates should be interpreted as preliminary benchmarks rather than definitive performance parameters. Despite these limitations, our study employed a rigorous methodology, including bivariate random-effects modeling, optimal cutoff analysis with the diagmeta method, and clinical utility assessment through Fagan nomograms and LR-based frameworks. The identification of an equivalence range rather than a single optimal cutoff represents a highly clinically meaningful approach when diagnostic performance remains stable across a range of thresholds.

## 5. Conclusions

CSF HBP demonstrates good overall diagnostic performance for HAVM and shows promise as a valuable adjunctive biomarker. Rather than recommending a single cutoff, we suggest a practical framework based on LR-derived thresholds: CSF HBP above 41.3 ng/mL (LR+ ≥10) for ruling in and below 30.1 ng/mL (LR− ≤0.1) for ruling out HAVM, with intermediate values requiring the integration of clinical context and underlying pathology. Although CSF HBP is not yet incorporated into routine diagnostic algorithms, our results provide quantitative data that may inform its future integration as an adjunctive tool in carefully selected clinical contexts. Prospective multicenter studies with standardized reference criteria and head-to-head biomarker comparisons are needed to validate our findings.

## Figures and Tables

**Table 1:** Subgroup and sensitivity analyses of the pooled diagnostic accuracy of cerebrospinal fluid heparin-binding protein for healthcare-associated ventriculitis and meningitis.

Analysis	k	*N*	Sensitivity (95% CI)	Specificity (95% CI)	LR+ (95% CI)	LR− (95% CI)	DOR (95% CI)
**Overall (all studies)**	**12**	**1761**	**0.861 (0.777–0.917)**	**0.848 (0.781–0.897)**	**5.65 (3.62–8.75)**	**0.164 (0.094–0.280)**	**34.5 (13.3–88.8)**
**Underlying pathology:** ICH-predominant	3	358	0.675 (0.556–0.775)	0.755 (0.688–0.812)	2.76 (2.12–3.59)	0.430 (0.302–0.584)	6.4 (3.8–10.9)
Non-ICH	9	1403	0.900 (0.842–0.939)	0.872 (0.794–0.923)	7.02 (4.18–12.13)	0.114 (0.067–0.193)	61.5 (22.5–168.3)
Brain tumor-predominant	3	511	0.935 (0.861–0.971)	0.922 (0.756–0.978)	11.99 (3.62–44.81)	0.070 (0.030–0.176)	170.8 (22.3–1334.7)
**Study design:** Prospective/partially prospective	4	869	0.896 (0.797–0.950)	0.912 (0.707–0.978)	10.18 (3.00–41.09)	0.114 (0.055–0.239)	89.4 (16.1–505.0)
Retrospective	8	892	0.849 (0.723–0.924)	0.833 (0.748–0.893)	5.08 (2.96–8.55)	0.181 (0.086–0.362)	28.0 (8.4–94.1)
**Assay method:** ELISA	5	561	0.788 (0.669–0.872)	0.760 (0.710–0.804)	3.28 (2.55–4.14)	0.279 (0.167–0.441)	11.8 (6.0–23.1)
Non-ELISA	6	1086	0.886 (0.763–0.949)	0.865 (0.774–0.923)	6.56 (3.54–12.09)	0.132 (0.056–0.296)	49.5 (12.7–194.8)
**Reference standard:** Strict (IDSA/CDC/microbiological)	5	976	0.905 (0.808–0.955)	0.868 (0.769–0.929)	6.87 (3.63–13.26)	0.110 (0.049–0.241)	62.5 (15.9–246.2)
Composite clinical criteria	7	785	0.820 (0.688–0.905)	0.830 (0.719–0.903)	4.83 (2.55–9.17)	0.216 (0.107–0.420)	22.3 (6.2–80.6)

**Abbreviations:** CI, confidence interval; CSF, cerebrospinal fluid; DOR, diagnostic odds ratio; ELISA, enzyme-linked immunosorbent assay; HBP, heparin-binding protein; ICH, intracerebral hemorrhage; IDSA, Infectious Diseases Society of America; CDC, Centers for Disease Control and Prevention; LR+, positive likelihood ratio; LR−, negative likelihood ratio. **Notes:** All analyses used bivariate random-effects models. k denotes the number of studies included in each analysis. Subgroups with k < 3 were not analyzed. 95% CIs for LR+, LR−, and DOR were derived via Monte Carlo simulation (100,000 draws).

## Data Availability

No new data were created or analyzed in this study. Data sharing is not applicable to this article.

## Supplementary Materials

The following supporting information can be downloaded at: https://www.mdpi.com/article/10.3390/diagnostics16071110/s1, Table S1: PRISMA-DTA checklist; Table S2: Complete search strategies for each database; Table S3: Characteristics of the 12 included studies; Table S4: QUADAS-3 quality assessment results; Figure S1: Predicted sensitivity and specificity as functions of the cutoff value; Figure S2: Predicted SROC curve from the diagmeta cutoff model; Figure S3: The Youden Index as a function of the cutoff value with a 95% bootstrap confidence interval.

## References

[1] Bischoff P., Schroder C., Gastmeier P., Geffers C. (2020). Surveillance of external ventricular drainage-associated meningitis and ventriculitis in German intensive care units. Infect. Control Hosp. Epidemiol..

[2] Ramanan M., Lipman J., Shorr A., Shankar A. (2015). A meta-analysis of ventriculostomy-associated cerebrospinal fluid infections. BMC Infect. Dis..

[3] Luque-Paz D., Revest M., Eugene F., Boukthir S., Dejoies L., Tattevin P., Le Reste P.J. (2021). Ventriculitis: A Severe Complication of Central Nervous System Infections. Open Forum Infect. Dis..

[4] Ramanan M., Shorr A., Lipman J. (2021). Ventriculitis: Infection or Inflammation. Antibiotics.

[5] Rogers T., Sok K., Erickson T., Aguilera E., Wootton S.H., Murray K.O., Hasbun R. (2019). Impact of Antibiotic Therapy in the Microbiological Yield of Healthcare-Associated Ventriculitis and Meningitis. Open Forum Infect. Dis..

[6] Tunkel A.R., Hasbun R., Bhimraj A., Byers K., Kaplan S.L., Scheld W.M., van de Beek D., Bleck T.P., Garton H.J.L., Zunt J.R. (2017). 2017 Infectious Diseases Society of America’s Clinical Practice Guidelines for Healthcare-Associated Ventriculitis and Meningitis. Clin. Infect. Dis..

[7] Linder A., Soehnlein O., Akesson P. (2010). Roles of heparin-binding protein in bacterial infections. J. Innate Immun..

[8] Gautam N., Olofsson A.M., Herwald H., Iversen L.F., Lundgren-Akerlund E., Hedqvist P., Arfors K.E., Flodgaard H., Lindbom L. (2001). Heparin-binding protein (HBP/CAP37): A missing link in neutrophil-evoked alteration of vascular permeability. Nat. Med..

[9] Linder A., Akesson P., Brink M., Studahl M., Bjorck L., Christensson B. (2011). Heparin-binding protein: A diagnostic marker of acute bacterial meningitis. Crit. Care Med..

[10] Kong Y., Ye Y., Ma J., Shi G. (2022). Accuracy of heparin-binding protein for the diagnosis of nosocomial meningitis and ventriculitis. Crit. Care.

[11] Pan X., Haishaer D., Liu M., Zhou S., Na H., Zhao H. (2024). Diagnostic, monitoring, and prognostic value of combined detection of cerebrospinal fluid heparin-binding protein, interleukin-6, interleukin-10, and procalcitonin for post-neurosurgical intracranial infection. Cytokine.

[12] McInnes M.D.F., Moher D., Thombs B.D., McGrath T.A., Bossuyt P.M., PRISMA-DTA, Clifford T., Cohen J.F., Deeks J.J., Gatsonis C. (2018). Preferred Reporting Items for a Systematic Review and Meta-analysis of Diagnostic Test Accuracy Studies: The PRISMA-DTA Statement. JAMA.

[13] Whiting P.F., Tomlinson E., Rutjes A.W.S., Davenport C.F., Yang B., Westwood M.E., Takwoingi Y., Reitsma J.B., Hyde C., Bossuyt P.M.M. (2026). QUADAS-3: A Revised Tool for the Quality Assessment of Diagnostic Test Accuracy Studies. Ann. Intern. Med..

[14] Reitsma J.B., Glas A.S., Rutjes A.W., Scholten R.J., Bossuyt P.M., Zwinderman A.H. (2005). Bivariate analysis of sensitivity and specificity produces informative summary measures in diagnostic reviews. J. Clin. Epidemiol..

[15] Fagan T.J. (1975). Letter: Nomogram for Bayes’s theorem. N. Engl. J. Med..

[16] Deeks J.J., Macaskill P., Irwig L. (2005). The performance of tests of publication bias and other sample size effects in systematic reviews of diagnostic test accuracy was assessed. J. Clin. Epidemiol..

[17] Steinhauser S., Schumacher M., Rucker G. (2016). Modelling multiple thresholds in meta-analysis of diagnostic test accuracy studies. BMC Med. Res. Methodol..

[18] Kong Y., Niu J., Liu Z., Zhou W., Tian Y., Shi G. (2025). Heparin-Binding Protein as a Diagnostic Biomarker for Healthcare-Associated Meningitis and Ventriculitis in Pediatric Patients. Neurosurgery.

[19] Zhang R., Huang Y., Deng Y., Chen D. (2022). Effects of intracranial infection of different types of pathogenic bacteria on the levels of NSE, NLRP3 and HBP in cerebrospinal fluid after craniotomy in elderly patients with cerebral hemorrhage. J. Mol. Diagn. Ther..

[20] Chen J., Qin H., Xie S., Hu Q. (2021). Changes and significance of HBP and HMGB-1 levels in cerebrospinal fluid of patients with secondary intracranial infection after craniocerebral surgery. Shandong Med. J..

[21] Guan S. (2023). Prediction value of heparin binding protein combined with procalcitonin in cerebrospinal fluid for intracranial infection after minimally invasive surgery for hypertensive intracerebral hemorrhage. Chin. Foreign Med. Res..

[22] Li X., Zhang X., He Z. (2021). Diagnostic value of cerebrospinal fluid PCT, CD64 and HBP combined detection in bacterial meningitis after neurosurgery. Chongqing Med..

[23] Liu J., Zhan Y., Liu X. (2021). Diagnostic value of serum and cerebrospinal fluid heparin-binding protein in early intracranial infection after craniotomy. Chin. J. Minim. Invasive Neurosurg..

[24] Wu T. (2025). Diagnostic value of cerebrospinal fluid neuron-specific enolase, heparin-binding protein and high mobility group protein B-1 for bacterial meningitis after brain tumor surgery. J. Chin. Foreign Med. Pharm. Res..

[25] Zhu X., Zhu W., Cheng X., Wu H. (2025). Predictive value of cerebrospinal fluid heparin-binding protein and CD64 expression for postoperative central nervous system infection following intracerebral hemorrhage surgery. Am. J. Transl. Res..

[26] Guan L., Wang F., Chen J., Xu Y., Zhang W., Zhu J. (2024). Clinical value of heparin-binding protein in adult bacterial intracranial infection. Front. Cell. Infect. Microbiol..

[27] Widen J., Cederberg D., Linder A., Westman G. (2023). Heparin-binding protein as a marker of ventriculostomy related infection and central nervous system inflammation in neuro-intensive care. Clin. Neurol. Neurosurg..

[28] Jaeschke R., Guyatt G.H., Sackett D.L. (1994). Users’ guides to the medical literature. III. How to use an article about a diagnostic test. B. What are the results and will they help me in caring for my patients? The Evidence-Based Medicine Working Group. JAMA.

[29] Zhang Y., Xiao X., Zhang J., Gao Z., Ji N., Zhang L. (2017). Diagnostic accuracy of routine blood examinations and CSF lactate level for post-neurosurgical bacterial meningitis. Int. J. Infect. Dis..

[30] Biasucci D.G., Sergi P.G., Bilotta F., Dauri M. (2024). Diagnostic Accuracy of Procalcitonin in Bacterial Infections of the CNS: An Updated Systematic Review, Meta-Analysis, and Meta-Regression. Crit. Care Med..

[31] Xiao X., Zhang Y., Zhang L., Kang P., Ji N. (2016). The diagnostic value of cerebrospinal fluid lactate for post-neurosurgical bacterial meningitis: A meta-analysis. BMC Infect. Dis..

[32] Olie S.E., Staal S.L., da Cruz Campos A.C., Bodilsen J., Nielsen H., van de Beek D., Brouwer M.C. (2025). Heparin-Binding Protein in Cerebrospinal Fluid as a Biomarker for Bacterial Meningitis: A Study of Diagnostic Accuracy. Ann. Neurol..

[33] Rutjes A.W., Reitsma J.B., Di Nisio M., Smidt N., van Rijn J.C., Bossuyt P.M. (2006). Evidence of bias and variation in diagnostic accuracy studies. Can. Med. Assoc. J..

