# Supplementary material for: Meta-Analytic Modeling to Define Decision Thresholds for Cerebrospinal Fluid Heparin-Binding Protein in Healthcare-Associated Ventriculitis and Meningitis
Source: Diagnostics (Basel). 2026 Apr 7;16(7):1110. doi: 10.3390/diagnostics16071110 (PMC13074188; doi:10.3390/diagnostics16071110)
Supplement: Supplementary file 1 [file diagnostics-16-01110-s001.zip › diagnostics-4217052-supplementary.pdf]

Table S1. PRISMA-DTA checklist for reporting of this diagnostic test accuracy systematic review and meta-analysis.

Section/topic	#	PRISMA-DTA Checklist Item	Reported on page #
**TITLE / ABSTRACT**			
Title	1	Identify the report as a systematic review (+/- meta-analysis) of diagnostic test accuracy (DTA) studies.	Title identifies report as a meta-analysis. The term "systematic review" appears in the Abstract (Background/Objectives) and Introduction.
Abstract	2	Abstract: See PRISMA-DTA for abstracts.	Abstract (structured: Background/Objectives, Methods, Results, Conclusions)
**INTRODUCTION**			
Rationale	3	Describe the rationale for the review in the context of what is already known.	Background, paragraphs 1–2: limitations of traditional CSF markers, mortality of HAVM, and emerging role of HBP
Clinical role of index test	D1	State the scientific and clinical background, including the intended use and clinical role of the index test, and if applicable, the rationale for minimally acceptable test accuracy.	Background, paragraphs 2–3: HBP mechanism, clinical role as adjunctive biomarker, and limitations of existing single-center studies
Objectives	4	Provide an explicit statement of question(s) being addressed in terms of participants, index test(s), and target condition(s).	Background, paragraph 4: " We therefore conducted the first systematic review and meta-analysis to determine the pooled diagnostic accuracy of CSF HBP for HAVM..."
**METHODS**			
Protocol and registration	5	Indicate if a review protocol exists, if and where it can be accessed (e.g., Web address), and, if available, provide registration information including registration number.	Registered in INPLASY (registration number: INPLASY202620068; DOI: 10.37766/inplasy2026.2.0068).
Eligibility criteria	6	Specify study characteristics (participants, setting, index test(s), reference standard(s), target condition(s), and study design) and report characteristics used as criteria for eligibility, giving rationale.	Methods > Eligibility Criteria: PIRD framework; participants, index test (CSF HBP), reference standard, study design, exclusion criteria
Information sources	7	Describe all information sources (e.g., databases with dates of coverage, contact with study authors to identify additional studies) in the search and date last searched.	Methods > Information Sources and Search Strategy: PubMed, Embase, Cochrane Library, CNKI; inception to February 15, 2026; reference lists of included studies
Search	8	Present full search strategies for all electronic databases and other sources searched, including any limits used, such that they could be repeated.	Table S1 (complete search strategies for all four databases)
Study selection	9	State the process for selecting studies (i.e., screening, eligibility, included in systematic review, and, if applicable, included in the meta-analysis).	Methods > Study Selection and Data Extraction: two independent reviewers, consensus resolution, PRISMA flow diagram (Figure 1)
Data collection process	10	Describe method of data extraction from reports (e.g., piloted forms, independently, in duplicate) and any processes for obtaining and confirming data from	Methods > Study Selection and Data Extraction: standardized spreadsheet, two independent reviewers, 2×2 contingency tables extracted

		investigators.	
Definitions for data extraction	11	Provide definitions used in data extraction and classifications of target condition(s), index test(s), reference standard(s) and other characteristics.	Methods > Eligibility Criteria: HAVM definitions (IDSA, CDC/NHSN, composite clinical criteria); Additional Table S3 (reference standard per study)
Risk of bias and applicability	12	Describe methods used for assessing risk of bias in individual studies and concerns regarding the applicability to the review question.	Methods > Quality Assessment: QUADAS-3 tool, two independent reviewers, four risk-of-bias domains and three applicability domains
Diagnostic accuracy measures	13	State the principal diagnostic accuracy measure(s) reported and state the unit of assessment.	Methods > Statistical Analysis: sensitivity, specificity, DOR, positive/negative likelihood ratios; per-patient assessment
Synthesis of results	14	Describe methods of handling data, combining results of studies and describing variability between studies.	Methods > Statistical Analysis: bivariate random-effects model (Reitsma), SROC curve, Cochran's Q, I^2, Spearman correlation for threshold effect, handling of multiple thresholds via diagmeta method
Meta-analysis	D2	Report the statistical methods used for meta-analyses, if performed.	Methods > Statistical Analysis: bivariate random-effects model, mada package in R v4.5.0; Deeks' funnel plot for publication bias
Additional analyses	16	Describe methods of additional analyses (e.g., sensitivity or subgroup analyses, meta-regression), if done, indicating which were pre-specified.	Methods > Statistical Analysis, paragraph 2: subgroup analyses by pathology, study design, assay method, reference standard; meta-regression with likelihood ratio test
**RESULTS**			
Study selection	17	Provide numbers of studies screened, assessed for eligibility, included in the review with reasons for exclusions at each stage, ideally with a flow diagram.	Results > Study Selection + Figure 1: 92 records identified (PubMed 46, Embase 11, Cochrane 0, CNKI 35), 6 duplicates removed, 86 screened, 69 excluded, 17 full-text assessed, 5 excluded (not DTA n=2, community-acquired n=1, plasma HBP n=2), 12 included
Study characteristics	18	For each included study provide citations and present key characteristics.	Results > Study Characteristics + Table S3: country, design, population, sample size, assay, cutoff, reference standard, Se/Sp
Risk of bias and applicability	19	Present evaluation of risk of bias and concerns regarding applicability for each study.	Results > Quality Assessment + Table S4: QUADAS-3 results per study; patient selection identified as primary concern (50% high risk of bias)
Results of individual studies	20	For each analysis in each study report 2×2 data with estimates of diagnostic accuracy and confidence intervals, ideally with a forest or ROC plot.	Additional Table S3 (2×2 data per study) + Figure 2 (paired forest plots of sensitivity and specificity with 95% CIs) + Figure 3 (SROC plot with individual study points and confidence/prediction regions)
Synthesis of results	21	Describe test accuracy, including variability; if meta-analysis was done, include results and confidence intervals.	Results > Diagnostic Accuracy: pooled Se 0.861 (95% CI: 0.777–0.917), Sp 0.848 (0.781–0.897), DOR 34.5 (13.3–88.8); heterogeneity: I^2 sensitivity 80.6%, specificity 98.5%
Additional analysis	23	Give results of additional analyses, if done.	Results > Subgroup and Meta-Regression + Table 1: ICH vs non-ICH pathology (significant, P=0.017); prospective vs retrospective; assay type; reference standard; Optimal Cutoff Analysis (28.4 ng/mL, equivalence range 20–50 ng/mL)
**DISCUSSION**			
Summary of evidence	24	Summarize the main findings including the strength of evidence.	Discussion, paragraph 1: first meta-analysis of CSF HBP for HAVM; good overall accuracy; clinical utility with dual-threshold approach
Limitations	25	Discuss limitations from included studies and from the	Discussion, paragraphs 4–5: retrospective designs, reference standard heterogeneity, ICH population

		review process.	performance, geographic concentration (China), single-center designs
Conclusions	26	Provide a general interpretation of the results in the context of other evidence. Discuss implications for future research and clinical practice.	Conclusion: CSF HBP as adjunctive biomarker; dual-threshold algorithm (rule-out ≤30.1 ng/mL, rule-in ≥41.3 ng/mL); need for multicenter prospective validation
**FUNDING**			
Funding	27	For the systematic review, describe the sources of funding and other support and the role of the funders.	Declarations > Funding: " This research received no external funding."

Table S2. Complete Search Strategies for Each Database

**PubMed (via MEDLINE)**	
**Line**	**Search Terms**
#1	"heparin-binding protein"[tiab] OR "heparin binding protein"[tiab] OR azurocidin[tiab] OR HBP[tiab] OR "CAP37"[tiab]
#2	"heparin-binding proteins"[MeSH Terms] OR "antimicrobial cationic peptides"[MeSH Terms]
#3	#1 OR #2
#4	ventriculitis[tiab] OR meningitis[tiab] OR ventriculomeningitis[tiab] OR "central nervous system infection*"[tiab] OR "intracranial infection*"[tiab] OR "CNS infection*"[tiab]
#5	"meningitis"[MeSH Terms] OR "central nervous system infections"[MeSH Terms] OR "ventriculitis"[MeSH Terms]
#6	#4 OR #5
#7	"healthcare-associated"[tiab] OR "health care associated"[tiab] OR nosocomial[tiab] OR "hospital-acquired"[tiab] OR postoperative[tiab] OR "post-operative"[tiab] OR postsurgical[tiab] OR "post-surgical"[tiab] OR "post-neurosurgical"[tiab] OR postcraniotomy[tiab] OR "post-craniotomy"[tiab]
#8	"external ventricular drain*"[tiab] OR "EVD"[tiab] OR "ventriculostomy"[tiab] OR "cerebrospinal fluid shunt*"[tiab] OR "CSF shunt*"[tiab] OR "ventriculoperitoneal shunt*"[tiab] OR craniotomy[tiab] OR neurosurg*[tiab]
#9	"neurosurgical procedures"[MeSH Terms] OR "craniotomy"[MeSH Terms] OR "ventriculostomy"[MeSH Terms] OR "cerebrospinal fluid shunts"[MeSH Terms] OR "cross infection"[MeSH Terms]
#10	#7 OR #8 OR #9
#11	"cerebrospinal fluid"[tiab] OR "CSF"[tiab] OR "spinal fluid"[tiab]
#12	"cerebrospinal fluid"[MeSH Terms]
#13	#11 OR #12
#14	#3 AND #6 AND #10 AND #13

Embase (via Elsevier)	
**Line**	**Search Terms**
#1	'heparin binding protein':ab,ti OR azurocidin:ab,ti OR 'HBP':ab,ti OR 'CAP37':ab,ti
#2	'heparin binding protein'/exp
#3	#1 OR #2
#4	ventriculitis:ab,ti OR meningitis:ab,ti OR ventriculomeningitis:ab,ti OR 'central nervous system infection*':ab,ti OR 'intracranial infection*':ab,ti OR 'CNS infection*':ab,ti
#5	'meningitis'/exp OR 'central nervous system infection'/exp OR 'ventriculitis'/exp
#6	#4 OR #5
#7	'healthcare associated':ab,ti OR nosocomial:ab,ti OR 'hospital acquired':ab,ti OR postoperative:ab,ti OR postsurgical:ab,ti OR 'post neurosurgical':ab,ti OR postcraniotomy:ab,ti
#8	'external ventricular drain*':ab,ti OR 'EVD':ab,ti OR ventriculostomy:ab,ti OR 'cerebrospinal fluid shunt*':ab,ti OR 'ventriculoperitoneal shunt*':ab,ti OR craniotomy:ab,ti OR neurosurg*:ab,ti
#9	'neurosurgery'/exp OR 'craniotomy'/exp OR 'brain ventricle drainage'/exp OR 'cerebrospinal fluid shunt'/exp OR 'hospital infection'/exp
#10	#7 OR #8 OR #9
#11	'cerebrospinal fluid':ab,ti OR 'CSF':ab,ti OR 'spinal fluid':ab,ti
#12	'cerebrospinal fluid'/exp
#13	#11 OR #12
#14	#3 AND #6 AND #10 AND #13

Cochrane Library	
**Line**	**Search Terms**

#1	("heparin-binding protein" OR "heparin binding protein" OR azurocidin OR HBP OR CAP37):ti,ab,kw
#2	[mh "heparin-binding proteins"]
#3	#1 OR #2
#4	(ventriculitis OR meningitis OR ventriculomeningitis OR "central nervous system infection*" OR "intracranial infection*" OR "CNS infection*"):ti,ab,kw
#5	[mh meningitis] OR [mh "central nervous system infections"] OR [mh ventriculitis]
#6	#4 OR #5
#7	("healthcare-associated" OR nosocomial OR "hospital-acquired" OR postoperative OR postsurgical OR "post-neurosurgical" OR postcraniotomy):ti,ab,kw
#8	("external ventricular drain*" OR EVD OR ventriculostomy OR "cerebrospinal fluid shunt*" OR craniotomy OR neurosurg*):ti,ab,kw
#9	[mh "neurosurgical procedures"] OR [mh craniotomy] OR [mh ventriculostomy] OR [mh "cerebrospinal fluid shunts"] OR [mh "cross infection"]
#10	#7 OR #8 OR #9
#11	("cerebrospinal fluid" OR CSF OR "spinal fluid"):ti,ab,kw
#12	[mh "cerebrospinal fluid"]
#13	#11 OR #12
#14	#3 AND #6 AND #10 AND #13

**China National Knowledge Infrastructure (CNKI)**	
**Line**	**Search Terms**
#1	SU=(“肝素结合蛋白” OR “heparin-binding protein” OR “azurocidin” OR “HBP”)
#2	SU=(“脑室炎” OR “脑膜炎” OR “中枢神经系统感染” OR “颅内感染”)
#3	SU=(“术后” OR “医院获得性” OR “开颅” OR “脑室引流” OR “分流” OR “神经外科”)

#4	SU=("脑脊液")
#5	#1 AND #2 AND #3 AND #4

Notes: The search strategy was structured around four key concepts combined with AND: (1) heparin-binding protein/azurocidin; (2) ventriculitis/meningitis/central nervous system infection; (3) healthcare-associated/nosocomial/post-neurosurgical context including external ventricular drains and cerebrospinal fluid shunts; and (4) cerebrospinal fluid. Both controlled vocabulary (MeSH/Emtree) and free-text terms were used. No language, date, or study design filters were applied.

Table S3. Characteristics of Included Studies on CSF Heparin-Binding Protein for Diagnosis of Healthcare-Associated Ventriculitis and Meningitis

Study	Study Design / Setting	Patient Population	Infected (n) / Control (n)	HBP Assay Method	Reference Standard	Cut-off (ng/mL)	Se	Sp
Li et al., 2021 *China*	Retrospective, single-center, Dept. of Neurosurgery 2017–2019	Post-neurosurgical patients with dura opening (craniotomy) *Population not specified by primary diagnosis*	38 / 20 (PAM)	Latex immunoturbidimetry	Clinical criteria + CSF parameters (Chinese Ministry of Health diagnostic criteria)	NR	0.90	0.75
Liu et al., 2021 *China*	Retrospective, single-center, Dept. of Neurosurgery Jan 2017–Dec 2019	Post-craniotomy patients with suspected early intracranial infection *Population not specified by primary diagnosis*	48 / 66	Not specified	Chinese Ministry of Health diagnostic criteria + CDC surgical site infection criteria	92.5	0.96	0.97
Chen et al., 2021 *China*	Retrospective, single-center, ICU Jan 2019–Feb 2021	Post-craniocerebral surgery patients *Population not specified by primary diagnosis*	58 / 83	ELISA	Chinese Ministry of Health diagnostic criteria for nosocomial infection	NR	0.81	0.76
Kong et al., 2022 *China*	Prospective, single-center, Dept. of Critical Care Medicine Aug 2020–Jun 2021	Post-craniotomy with dura opening; predominantly CNS tumors (~76%), cerebrovascular disease, epilepsy, others	131 / 150	Fluorescence immunoassay	IDSA 2017 HAVM guidelines + CSF culture / clinical criteria	23.0	0.97	0.95
Zhang et al., 2022 *China*	Retrospective, single-center, Dept. of Critical Care Medicine Aug 2019–Oct 2021	Exclusively elderly post-ICH craniotomy patients	35 / 81	ELISA	Clinical criteria + CSF parameters	30.73	0.63	0.78
Widén et al., 2023 *Sweden*	Retrospective analysis of prospectively collected data, single-center, Neuro-ICU Jan 2009–Mar 2010	EVD patients; SAH 48%, TBI 27%, ICH 6%, shunt dysfunction 7%, others	7 / 75	ELISA	Positive CSF culture/PCR + CSF pleocytosis (leukocytes >50 × 10^6/L)	25.15	0.71	0.86
Guan et al., 2023 *China*	Retrospective, single-center Jan 2019–Nov 2022	Exclusively hypertensive ICH patients undergoing minimally invasive surgery	34 / 74	ELISA	Clinical criteria + CSF parameters (Chinese diagnostic criteria)	21.39	0.79	0.72
Pan et al., 2024 *China*	Retrospective, single-center Apr 2021–Apr 2022	Post-craniotomy patients with suspected intracranial infection *Population not specified by primary diagnosis*	53 / 54	Latex immunoturbidimetry	IDSA 2017 HAVM guidelines + CSF culture / clinical criteria	47.50	0.98	0.90
Guan et al., 2024 *China*	Prospective, single-center Nov 2021–Nov 2023	Post-neurosurgical (dura opening); trauma 38%, tumor 31%, ICH 21%, arachnoid cyst 10%	270 / 120	Latex immunoturbidimetry	CSF culture confirmed + clinical criteria; NGS for discrepant cases	15.80	0.85	1.00
Kong et al., 2025 *China*	Prospective, single-center, Pediatric Neurosurgical ICU Feb 2021–Feb 2023	Exclusively pediatric patients with craniotomy for ventricular system tumors (germ cell tumor, craniopharyngioma, glioma, ependymoma, others)	71 / 45	Fluorescence immunoassay	CDC/NHSN definition for meningitis/ventriculitis	74.0	0.89	0.81
Wu et al., 2025 *China*	Retrospective, single-center Jan 2023–Oct 2024	Exclusively post-brain tumor surgery patients	32 / 82 (PAM)	ELISA	Clinical criteria for bacterial meningitis	14.96	0.94	1.00
Zhu et al., 2025 *China*	Retrospective, single-center, Dept. of Neurosurgery Feb 2021–Feb 2024	Exclusively hypertensive ICH surgery patients	64 / 70	Fluorescence immunochromatography	Clinical criteria + CSF parameters	21.37	0.63	0.77

Abbreviations: Se, sensitivity; Sp, specificity; PAM, post-neurosurgical aseptic meningitis; ICH, intracerebral hemorrhage; HICH, hypertensive intracerebral hemorrhage; EVD, external ventricular drain; SAH, subarachnoid hemorrhage; TBI, traumatic brain injury; ELISA, enzyme-linked immunosorbent assay; IDSA, Infectious Diseases Society of America; HAVM, healthcare-associated ventriculitis and meningitis; CDC, Centers for Disease Control and Prevention; NHSN, National Healthcare Safety Network; NGS, next-generation sequencing; NR, not reported.

Table S4. Quality assessment of included studies using QUADAS-3

Study	Risk of Bias					Applicability Concerns			
	D1	D2	D3	D4	Overall	D1	D2	D3	Overall
Chen 2021	-	+	+	+	**-**	+	+	+	**+**
Guan 2023	-	+	?	+	**-**	+	+	+	**+**
Guan 2024	+	+	+	+	**+**	+	+	+	**+**
Kong 2022	+	+	+	+	**+**	+	+	+	**+**
Kong 2025	+	+	+	+	**+**	+	+	+	**+**
Li 2021	-	+	+	+	**-**	+	+	+	**+**
Liu 2021	+	?	+	+	**+**	+	+	+	**+**
Pan 2024	+	+	+	+	**+**	+	+	+	**+**
Widén 2023	+	+	+	+	**+**	+	+	+	**+**
Wu 2025	-	+	?	+	**-**	+	+	+	**+**
Zhang 2022	-	+	+	+	**-**	?	+	+	**?**
Zhu 2025	-	+	+	+	**-**	?	+	+	**?**

Risk of Bias Domains: D1 = Participants (patient selection); D2 = Index Test (CSF HBP measurement); D3 = Target Condition (reference standard); D4 = Analysis (flow and timing)

Applicability Domains: D1 = Participants; D2 = Index Test; D3 = Target Condition

Judgment symbols: + = Low risk/Low concern (green); - = High risk/High concern (red); ? = Unclear/Some concerns (yellow)

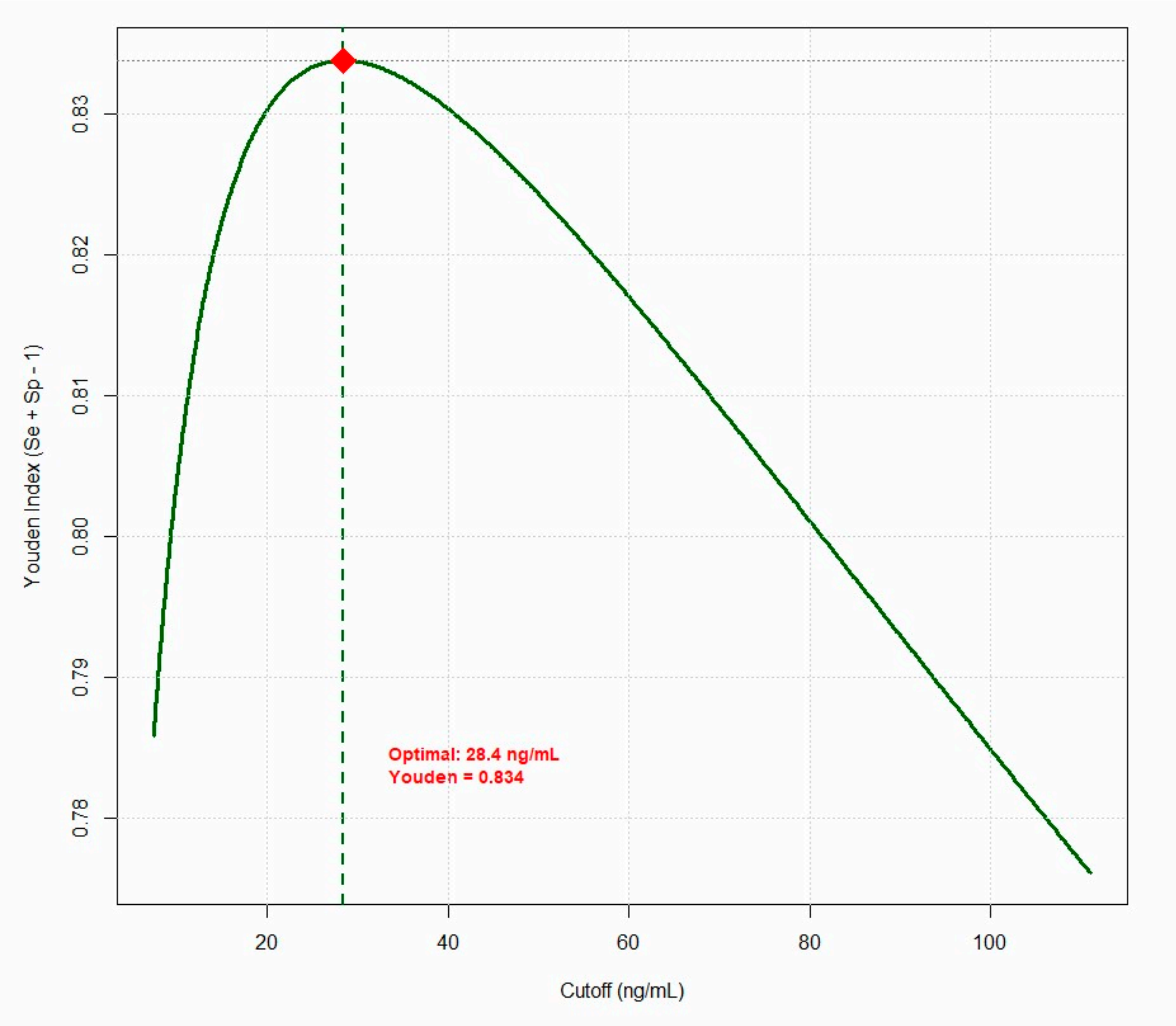

Figure S1. Youden index as a function of cerebrospinal fluid heparin-binding protein cutoff values. The optimal cutoff maximizing the Youden index was 28.4 ng/mL (Youden index = 0.834, red diamond). The dashed green line indicates the optimal cutoff. Se, sensitivity; Sp, specificity.

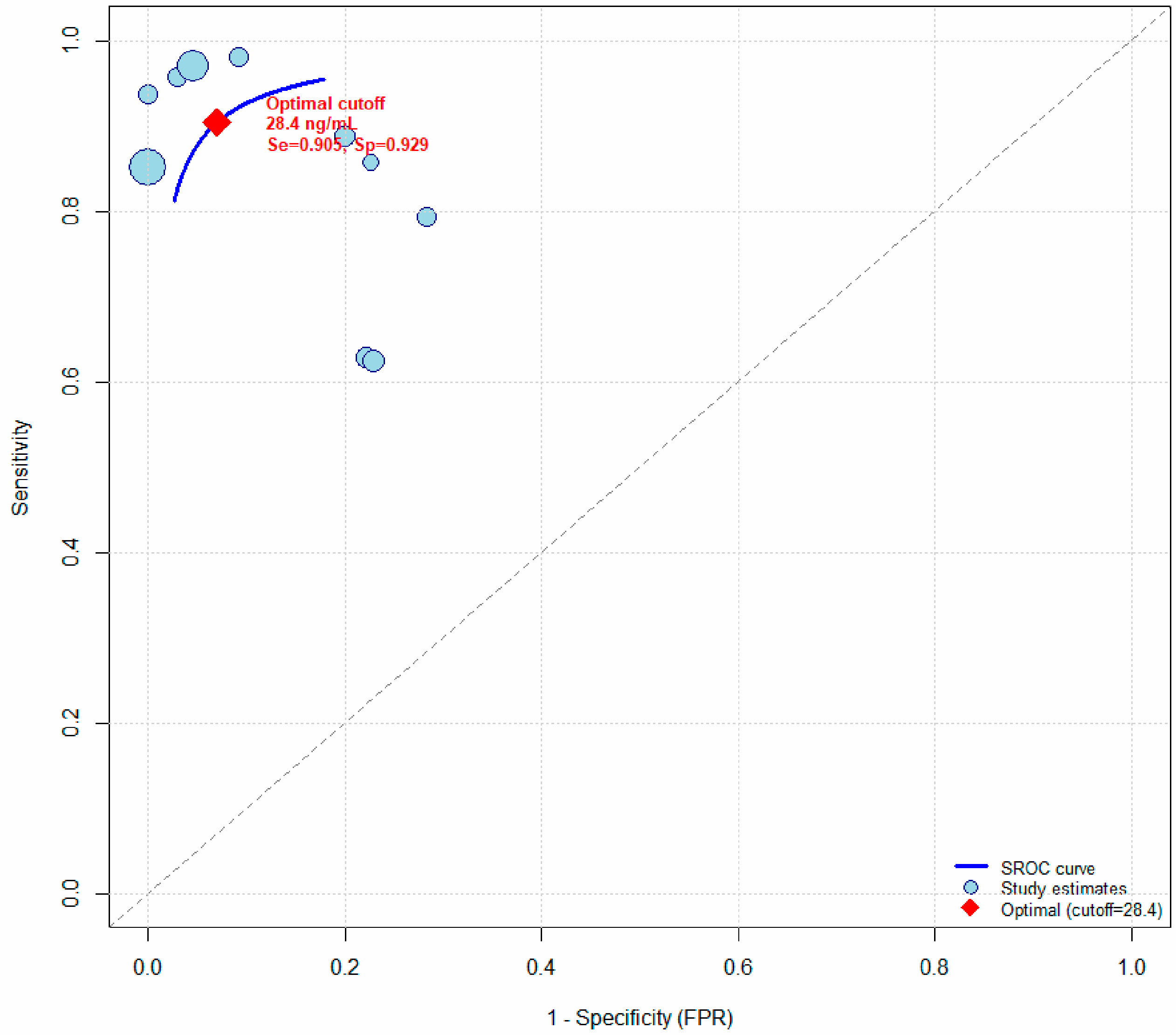

Figure S2. Summary receiver operating characteristic (SROC) curve derived from the diagmeta optimal cutoff analysis. Each circle represents an individual study, with circle size proportional to sample size. The red diamond indicates the predicted sensitivity (0.905) and specificity (0.929) at the optimal cutoff of 28.4 ng/mL. The diagonal gray line represents the line of no discrimination. FPR, false-positive rate; Se, sensitivity; Sp, specificity; SROC, summary receiver operating characteristic.

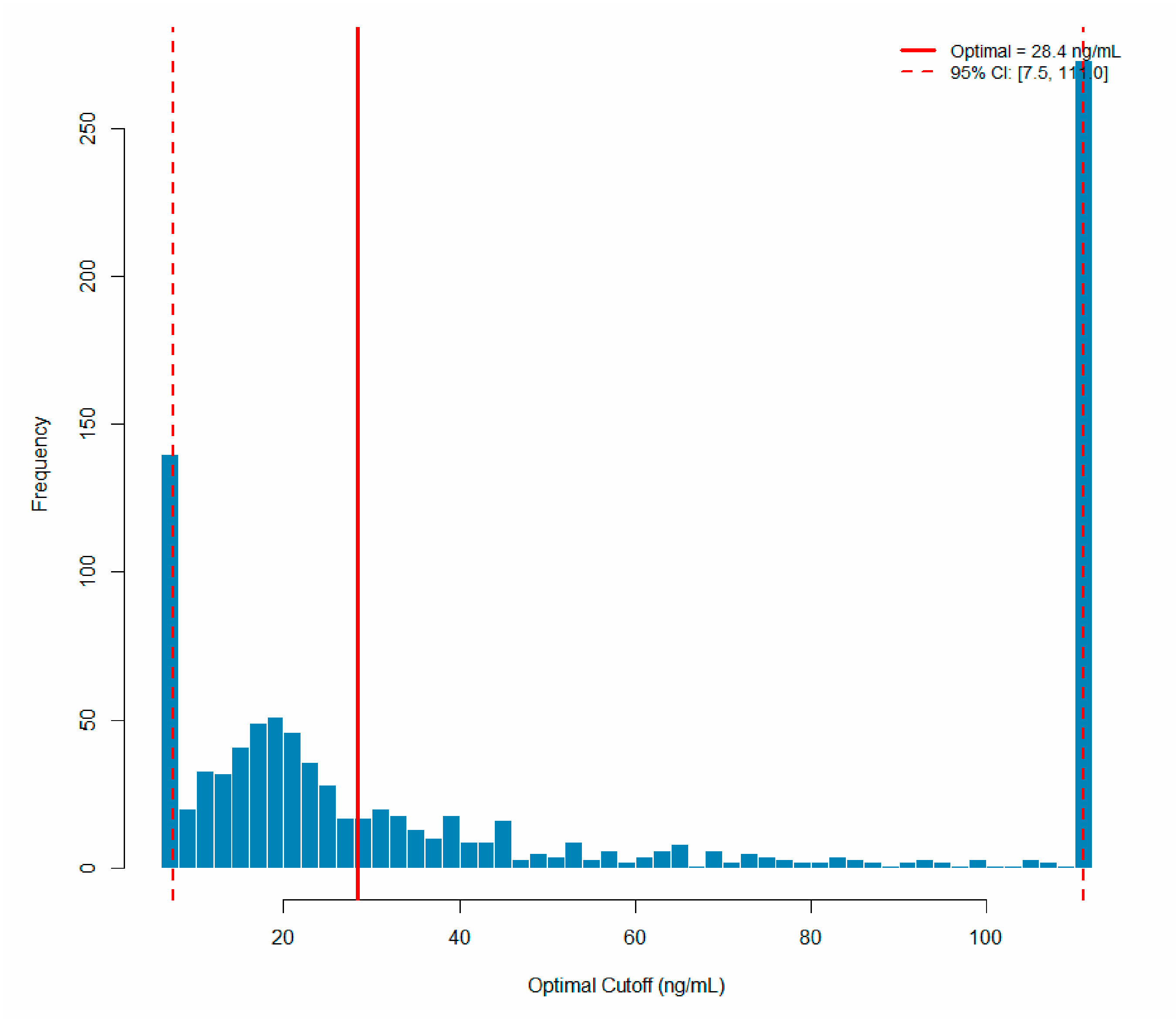

Figure S3. Bootstrap distribution of the optimal cutoff value from 1,000 resamples. The solid red line indicates the point estimate of the optimal cutoff (28.0 ng/mL). The dashed red lines indicate the 95% bootstrap confidence interval (7.5–111.0 ng/mL). The wide confidence interval and right-skewed distribution reflect substantial uncertainty in the optimal cutoff, supporting the use of an equivalence range rather than a single threshold. CI, confidence interval.
